# Supplementary material for: Undiagnosed hypertension and its associated factors in India: A rural-urban contrast from the National Family Health Survey (2019-21)
Source: PLoS One. 2025 May 21;20(5):e0316782. doi: 10.1371/journal.pone.0316782 (PMC12094755; doi:10.1371/journal.pone.0316782)
Supplement: S1 Table — (DOCX) [file pone.0316782.s001.docx]

Supplementary Table S1: Factors associated with undiagnosed hypertension (adjusted odds ratio with 95 % confidence intervals) among men and women aged 15-49, India, NFHS-5, 2019-21

| **Variables** | **Adjusted Odd ratio of undiagnosed hypertension among male** | | | **Adjusted Odd ratio of undiagnosed hypertension among female** | | |
| --- | --- | --- | --- | --- | --- | --- |
|  | **Total** | **Rural** | **Urban** | **Total** | **Rural** | **Urban** |
| **Age group** |  |  |  |  |  |  |
| 15-19® |  |  |  |  |  |  |
| 20-29 | 1.48***(1.21-1.8) | 1.61***(1.28-2.03) | 1.05 (0.7-1.58) | 0.98 (0.92-1.06) | 1.02 (0.94-1.1) | 0.91 (0.79-1.04) |
| 30-39 | 1.47***(1.18-1.82) | 1.62***(1.26-2.08) | 1.01 (0.65-1.57) | 1.32***(1.22-1.42) | 1.38***(1.27-1.51) | 1.12 (0.96-1.29) |
| 40-49 | 1.13 (0.91-1.4) | 1.28*(0.99-1.65) | 0.73 (0.47-1.13) | 1.34***(1.25-1.45) | 1.47***(1.35-1.6) | 1.02 (0.88-1.19) |
| **Anaemia** |  |  |  |  |  |  |
| Severe® |  |  |  |  |  |  |
| Moderate | 1.76*(0.93-3.36) | 1.88*(0.95-3.73) | 1.1 (0.14-8.8) | 1.51***(1.38-1.65) | 1.48***(1.34-1.64) | 1.58***(1.32-1.88) |
| Mild | 2.06**(1.1-3.86) | 2.19**(1.13-4.25) | 1.29 (0.17-10.06) | 1.62***(1.48-1.77) | 1.58***(1.43-1.75) | 1.71***(1.43-2.04) |
| Not anaemic | 2.49***(1.34-4.63) | 2.55***(1.33-4.93) | 1.69 (0.22-13.12) | 1.9***(1.74-2.07) | 1.85***(1.67-2.04) | 2.04***(1.72-2.43) |
| **BMI** |  |  |  |  |  |  |
| Underweight® |  |  |  |  |  |  |
| Normal weight | 1.18**(1.03-1.36) | 1.2**(1.03-1.4) | 1.09 (0.79-1.5) | 1.14***(1.09-1.19) | 1.14***(1.09-1.2) | 1.17***(1.05-1.3) |
| Overweight | 1.26***(1.08-1.46) | 1.33***(1.12-1.58) | 1.08 (0.77-1.5) | 1.26***(1.2-1.32) | 1.25***(1.18-1.32) | 1.32***(1.18-1.47) |
| Obese | 1.03 (0.85-1.23) | 1.1 (0.88-1.38) | 0.88 (0.61-1.27) | 1.07**(1.02-1.13) | 1.08**(1.01-1.15) | 1.1 (0.98-1.23) |
| **Waist to Hip Ratio** |  |  |  |  |  |  |
| Less or equal to 0.9 for male and 0.8 for female® |  |  |  |  |  |  |
| (Cut offs > 0.9 for males and >0.8 for females) | 1.08**(1-1.16) | 1.04 (0.96-1.14) | 1.18**(1.03-1.35) | 1.12***(1.09-1.15) | 1.1***(1.06-1.13) | 1.19***(1.13-1.25) |
| **Marital Status** |  |  |  |  |  |  |
| Never in union® |  |  |  |  |  |  |
| Currently married | 0.81***(0.72-0.91) | 0.81***(0.7-0.94) | 0.81*(0.65-1) | 0.63***(0.59-0.66) | 0.6***(0.56-0.64) | 0.68***(0.62-0.76) |
| Widowed/Divorced/Separated | 0.78*(0.59-1.03) | 0.86 (0.62-1.2) | 0.6*(0.34-1.04) | 0.6***(0.56-0.65) | 0.58***(0.53-0.63) | 0.67***(0.59-0.77) |
| **Education** |  |  |  |  |  |  |
| No education/Pre-primary education® |  |  |  |  |  |  |
| Primary | 1.06 (0.92-1.22) | 1.08 (0.93-1.27) | 1.05 (0.77-1.43) | 0.9***(0.87-0.94) | 0.89***(0.85-0.93) | 0.98 (0.9-1.07) |
| Secondary Education | 0.94 (0.83-1.05) | 0.92 (0.81-1.05) | 1.06 (0.81-1.37) | 0.82***(0.8-0.85) | 0.82***(0.79-0.85) | 0.88***(0.82-0.94) |
| Higher Education | 0.85**(0.73-0.99) | 0.81**(0.67-0.96) | 1.06 (0.78-1.43) | 0.78***(0.74-0.82) | 0.76***(0.71-0.81) | 0.85***(0.78-0.93) |
| **Cast/Tribe** |  |  |  |  |  |  |
| Scheduled caste® |  |  |  |  |  |  |
| Scheduled tribe | 1.42***(1.25-1.62) | 1.53***(1.32-1.77) | 0.91 (0.69-1.21) | 1.81***(1.73-1.89) | 1.88***(1.79-1.98) | 1.39***(1.25-1.55) |
| OBC | 1.06 (0.96-1.18) | 1.05 (0.93-1.18) | 1.09 (0.91-1.32) | 1.11***(1.07-1.15) | 1.12***(1.08-1.17) | 1.05 (0.98-1.13) |
| Others | 1.03 (0.93-1.15) | 1.05 (0.92-1.2) | 0.98 (0.81-1.2) | 1.09***(1.05-1.13) | 1.08***(1.03-1.13) | 1.1***(1.03-1.19) |
| **Religion** |  |  |  |  |  |  |
| Hindu® |  |  |  |  |  |  |
| Muslim | 0.71***(0.63-0.81) | 0.61***(0.52-0.71) | 0.97 (0.79-1.19) | 0.81***(0.78-0.84) | 0.76***(0.72-0.8) | 0.93**(0.86-0.99) |
| Christian | 0.92 (0.79-1.08) | 0.95 (0.79-1.14) | 1.02 (0.75-1.38) | 0.82***(0.77-0.87) | 0.84***(0.79-0.9) | 0.81***(0.72-0.91) |
| Others | 0.81***(0.71-0.94) | 0.83**(0.7-0.97) | 0.81 (0.6-1.09) | 0.81***(0.77-0.85) | 0.82***(0.77-0.87) | 0.81***(0.73-0.91) |
| **Wealth Index** |  |  |  |  |  |  |
| Poorest® |  |  |  |  |  |  |
| Poorer | 0.96 (0.85-1.08) | 0.98 (0.87-1.11) | 0.78 (0.47-1.3) | 0.87***(0.83-0.9) | 0.87***(0.84-0.91) | 0.87*(0.74-1.03) |
| Middle | 0.93 (0.83-1.06) | 0.96 (0.84-1.1) | 0.73 (0.45-1.18) | 0.82***(0.78-0.85) | 0.83***(0.79-0.87) | 0.81***(0.69-0.95) |
| Richer | 0.92 (0.8-1.05) | 0.98 (0.84-1.15) | 0.68 (0.42-1.1) | 0.78***(0.74-0.81) | 0.79***(0.75-0.83) | 0.77***(0.66-0.9) |
| Richest | 0.88 (0.76-1.02) | 0.96 (0.8-1.16) | 0.6**(0.37-0.99) | 0.76***(0.72-0.8) | 0.75***(0.71-0.8) | 0.73***(0.63-0.86) |
| **Occupation** |  |  |  |  |  |  |
| No Occupation® |  |  |  |  |  |  |
| Professional/ Clarical | 0.82**(0.68-1) | 0.82 (0.64-1.05) | 0.84 (0.61-1.16) |  |  |  |
| Sales | 0.9 (0.75-1.08) | 0.85 (0.67-1.07) | 0.94 (0.69-1.28) |  |  |  |
| Services/household and domestic | 0.82**(0.68-0.99) | 0.88 (0.69-1.12) | 0.78 (0.56-1.08) |  |  |  |
| Agricultural | 0.85*(0.72-1) | 0.83*(0.68-1) | 0.89 (0.62-1.28) |  |  |  |
| Skilled, unskilled manual and Others | 0.85*(0.72-1) | 0.84*(0.69-1.02) | 0.87 (0.65-1.17) |  |  |  |
| **Work at home or away** |  |  |  |  |  |  |
| At home® |  |  |  |  |  |  |
| Away | 0.78***(0.71-0.86) | 0.75***(0.68-0.84) | 0.87 (0.72-1.05) |  |  |  |
| **Use of Internet** |  |  |  |  |  |  |
| Never® |  |  |  |  |  |  |
| Yes | 0.94 (0.87-1.03) | 0.98 (0.89-1.08) | 0.85*(0.72-1.01) |  |  |  |
| **Owns a mobile telephone** |  |  |  |  |  |  |
| No® |  |  |  |  |  |  |
| Yes | 0.85**(0.74-0.98) | 0.8***(0.69-0.94) | 1.32 (0.92-1.88) |  |  |  |
| **Current smokeless tobacco user** |  |  |  |  |  |  |
| No® |  |  |  |  |  |  |
| Yes | 1.18***(1.06-1.32) | 1.11 (0.98-1.26) | 1.32***(1.08-1.62) | 0.85**(0.74-0.98) | 0.87*(0.75-1.02) | 0.77 (0.56-1.07) |
| **Usage any tobacco** |  |  |  |  |  |  |
| No® |  |  |  |  |  |  |
| Yes | 0.84***(0.76-0.93) | 0.89*(0.79-1.01) | 0.77***(0.64-0.92) | 1.07 (0.94-1.22) | 1.04 (0.9-1.21) | 1.21 (0.89-1.65) |
| **Alcohol usage current** |  |  |  |  |  |  |
| No® |  |  |  |  |  |  |
| Less than once a week | 1.11*(1-1.24) | 1.06 (0.93-1.21) | 1.23**(1-1.52) | 1.18***(1.05-1.32) | 1.19***(1.05-1.35) | 1.02 (0.78-1.32) |
| Once a week | 1.26***(1.13-1.39) | 1.23***(1.09-1.39) | 1.27**(1.04-1.54) | 1.23***(1.09-1.38) | 1.21***(1.07-1.37) | 1.08 (0.76-1.53) |
| Everyday | 1.08 (0.93-1.25) | 1.08 (0.91-1.28) | 1.03 (0.78-1.36) | 1.43***(1.18-1.73) | 1.39***(1.13-1.7) | 1.46 (0.8-2.65) |
| **Covered by Health Insurance** |  |  |  |  |  |  |
| No® |  |  |  |  |  |  |
| Yes | 1.01 (0.94-1.09) | 1.1**(1.01-1.2) | 0.81***(0.71-0.93) | 1.07***(1.05-1.1) | 1.08***(1.05-1.12) | 1.03 (0.98-1.08) |
| **Type of health facility recently use** |  |  |  |  |  |  |
| None® |  |  |  |  |  |  |
| Public facility | 0.69***(0.64-0.76) | 0.68***(0.62-0.76) | 0.74***(0.63-0.87) | 0.73***(0.71-0.76) | 0.72***(0.7-0.75) | 0.78***(0.73-0.83) |
| Private facility | 0.65***(0.58-0.72) | 0.67***(0.58-0.76) | 0.61***(0.5-0.73) | 0.64***(0.61-0.66) | 0.61***(0.59-0.64) | 0.69***(0.65-0.74) |
| Other | 0.89 (0.62-1.27) | 0.87 (0.59-1.28) | 0.98 (0.41-2.35) | 0.6***(0.48-0.73) | 0.56***(0.43-0.71) | 0.7*(0.47-1.05) |
| **Region** |  |  |  |  |  |  |
| North® |  |  |  |  |  |  |
| Central | 1.16**(1.03-1.3) | 1.2**(1.04-1.38) | 1.23*(0.99-1.53) | 1.35***(1.3-1.4) | 1.38***(1.31-1.44) | 1.32***(1.23-1.43) |
| East | 0.58***(0.51-0.67) | 0.63***(0.54-0.74) | 0.5***(0.39-0.65) | 0.83***(0.79-0.87) | 0.87***(0.82-0.92) | 0.74***(0.67-0.82) |
| Northeast | 0.57***(0.49-0.65) | 0.57***(0.49-0.68) | 0.6***(0.46-0.78) | 0.93***(0.88-0.98) | 0.96 (0.91-1.03) | 0.9**(0.8-1) |
| West | 1.45***(1.24-1.68) | 1.47***(1.21-1.77) | 1.42***(1.1-1.84) | 1.86***(1.77-1.96) | 1.98***(1.86-2.11) | 1.6***(1.47-1.75) |
| South | 1.14**(1-1.29) | 1.24***(1.06-1.45) | 0.96 (0.78-1.19) | 1.74***(1.66-1.83) | 1.79***(1.69-1.9) | 1.64***(1.51-1.78) |
| **Dietary Intake** |  |  |  |  |  |  |
| **Milk/curd** |  |  |  |  |  |  |
| (never/occasionally)® |  |  |  |  |  |  |
| (daily/weekly) | 1.09**(1.02-1.18) | 1.14***(1.04-1.24) | 0.97 (0.84-1.12) | 1.04***(1.02-1.07) | 1.03**(1-1.06) | 1.07***(1.02-1.12) |
| **Pulses/beans** |  |  |  |  |  |  |
| (never/occasionally)® |  |  |  |  |  |  |
| (daily/weekly) | 1.05 (0.97-1.13) | 1.07 (0.98-1.17) | 1 (0.86-1.15) | 0.99 (0.96-1.02) | 1.03*(1-1.06) | 0.89***(0.85-0.94) |
| **Dark green leafy vegetable** |  |  |  |  |  |  |
| (never/occasionally)® |  |  |  |  |  |  |
| (daily/weekly) | 1.02 (0.94-1.1) | 1 (0.92-1.1) | 1.05 (0.91-1.22) | 1 (0.98-1.03) | 0.99 (0.96-1.02) | 1.04 (0.99-1.1) |
| **Fruits** |  |  |  |  |  |  |
| (never/occasionally)® |  |  |  |  |  |  |
| (daily/weekly) | 1.21***(1.09-1.34) | 1.3***(1.14-1.48) | 1.06 (0.89-1.26) | 1.03 (0.99-1.07) | 1.08***(1.03-1.13) | 0.98 (0.92-1.04) |
| **Eggs** |  |  |  |  |  |  |
| (never/occasionally)® |  |  |  |  |  |  |
| (daily/weekly) | 1 (0.89-1.11) | 0.89*(0.78-1.02) | 1.23**(1.02-1.48) | 1.11***(1.06-1.15) | 1.12***(1.07-1.17) | 1.08**(1.01-1.15) |
| **Fish** |  |  |  |  |  |  |
| (never/occasionally)® |  |  |  |  |  |  |
| (daily/weekly) | 1.01 (0.91-1.13) | 1.07 (0.94-1.23) | 0.88 (0.72-1.07) | 1.11***(1.07-1.15) | 1.09***(1.04-1.15) | 1.13***(1.06-1.22) |
| **Chicken or meat** |  |  |  |  |  |  |
| (never/occasionally)® |  |  |  |  |  |  |
| (daily/weekly) | 1.01 (0.89-1.14) | 1.08 (0.92-1.26) | 0.9 (0.71-1.13) | 1 (0.96-1.05) | 1.01 (0.96-1.07) | 0.97 (0.89-1.05) |
| **Fried food** |  |  |  |  |  |  |
| (never/occasionally)® |  |  |  |  |  |  |
| (daily/weekly) | 1.09*(1-1.19) | 1.11*(1-1.23) | 1 (0.85-1.18) | 1.09***(1.05-1.13) | 1.07***(1.02-1.11) | 1.11***(1.04-1.19) |
| **Aerated drinks** |  |  |  |  |  |  |
| (never/occasionally)® |  |  |  |  |  |  |
| (daily/weekly) | 1.13***(1.04-1.24) | 1.06 (0.96-1.18) | 1.33***(1.13-1.56) | 0.96***(0.93-0.99) | 0.96**(0.92-0.99) | 0.97 (0.92-1.03) |
| Total N | 16474 | 11780 | 4694 | 116033 | 85410 | 30623 |
