# Supplementary material for: Undiagnosed hypertension and its associated factors in India: A rural-urban contrast from the National Family Health Survey (2019-21)
Source: PLoS One. 2025 May 21;20(5):e0316782. doi: 10.1371/journal.pone.0316782 (PMC12094755; doi:10.1371/journal.pone.0316782)
Supplement: Attachment — Submitted filename: Response to Reviewers PLOS ONE.docx [file pone.0316782.s002.docx]

**Response to Reviewers**

| **S. No.** | **Comments** | **Response** |
| --- | --- | --- |
| 1. | Please ensure that your manuscript meets PLOS ONE's style requirements, including those for file naming. | Changes have been made to meet the style requirements of PLOS ONE. |
| 2. | Thank you for uploading your study's underlying data set. Unfortunately, the repository you have noted in your Data Availability statement does not qualify as an acceptable data repository according to PLOS's standards. | The dataset is freely available from DHS sites (<https://dhsprogram.com/data/dataset/India_Standard-DHS_2020.cfm?flag=0>) as well as on reasonable request from DHS. It is also available from India’s Open Government Data platform - <https://www.data.gov.in/catalog/national-family-health-survey-nfhs-5>. |
| 3. | We note that Figures 2 and 3 in your submission contain map images which may be copyrighted. | I have received permission for usage from the software/website where I generated the figures. They have also confirmed that the image copyright belongs to me, rather than the site itself. They just need to be cited in the references. I have attached the email copy in “other files” |
| 4. | The conclusion have been drawn from a dataset which is inherently limited in the way data is collected and definitions/classifications have been established. This restricts the validity of the analysis performed. | The same has been further clarified in the limitations section. |
| 5. | Clearly emphasize that no causal inferences can be made and that further longitudinal studies are required which was missed out in the manuscript. | Have clarified in the limitations section. |
| 6. | Include a limitations section explicitly discussing unmeasured confounders and suggest future studies incorporating these factors. | Have added the same to limitations. |
| 7. | Acknowledge the possible effect of COVID-19 on undiagnosed hypertension in the discussion. | This has been acknowledged in the discussion section. |
| 8. | Justify the choice of the 140/90 mmHg cutoff and discuss how using 130/80 mmHg might have influenced prevalence estimates. | This has been clarified further in the methods and limitations. |
| 9. | Chi square test is needed for spatial variations which was not done. | This has been addressed in the limitations section. |
